# Morphological and molecular taxonomy of *Nidularia balachowskii* Bodenheimer (Hemiptera, Coccoidea, Kermesidae) with notes on its life history in Israel

**DOI:** 10.3897/zookeys.254.3959

**Published:** 2012-12-21

**Authors:** Malkie Spodek, Yair Ben-Dov, Murad Ghanim, Zvi Mendel

**Affiliations:** 1 Department of Entomology, Volcani Center, Agricultural Research Organization, POB 6, Bet Dagan 50250, Israel; 2 Department of Entomology, Robert H. Smith Faculty of Agriculture, Food and Environment, The Hebrew University of Jerusalem, POB 12, Rehovot 76100, Israel

**Keywords:** Scale insect, *Quercus* spp., morphology, univoltine, monophagous, 28S, COI

## Abstract

Descriptions and illustrations of the adult female and first-instar nymph of the kermesid *Nidularia balachowskii* Bodenheimer, based on the adult female lectotype and paralectotype (here designated), and new material collected from Israel are presented. A key for the identification of first-instar nymphs of *Nidularia* spp. is offered. Molecular identification of *Nidularia balachowskii*, using nucleotide sequences from the D2–D3 region of the 28S ribosomal gene, and the mitochondrial Cytochrome Oxidase I (COI) gene, is presented. Morphological and molecular analyses confirm that *Nidularia balachowskii* is closely related to other species within the Kermesidae. In Israel, this species develops only on *Quercus ithaburensis* and is univoltine. This is the first detailed report of *Nidulariabalachowskii* from Israel.

## Introduction

*Nidularia* Targioni-Tozzetti, 1868 is one of ten genera of scale insects within the Kermesidae (Hemiptera: Coccoidea). Three species of *Nidularia*: *Nidularia balachowskii* Bodenheimer, 1941, *Nidularia japonica* Kuwana, 1918 and *Nidularia pulvinata* (Planchon, 1864), have been recorded so far only from the Palaearctic region (Ben-Dov et al. 2012). *Nidularia pulvinata*, the type species of this genus (Signoret 1875, Morrison and Morrison 1966), occurs in western territories of the Palaearctic region, where it has been collected on three oak species, *Quercus coccifera* L., *Quercus ilex* L.and *Quercus ithaburensis* Decne. (Koteja 1980, Bullington and Kosztarab 1985).

The three species of *Nidularia* have been recorded as follows: *Nidularia pulvinata* from Algeria (Koteja 1980); China (Tang 1984); France, including Corsica (Balachowsky 1933, Foldi 2001, 2003); Italy (Hoy 1963, Viggiani 1991); Portugal (Hoy 1963); Spain (Gomez-Menor Ortega 1937, Hoy 1963); *Nidularia japonica* in China and Japan, on the branches and trunks of several oak species namely, *Quercus acutissima* Carruth., *Quercus ailena* Blume, *Quercus dentate* Thunb., *Quercus fabri* Hance., *Quercus glandulfera* Blume(Liu et al. 1997); while *Nidularia balachowskii* has been reported from Turkey (Bodenheimer 1941), Iran and Israel (Bodenheimer 1944) on *Quercus* sp..

The family placement of *Nidularia* has changed over the years. Targioni-Tozzetti (1868) placed this genus in the Coccidae *sensu lato*. Signoret (1875) and Koteja (1974, 1980) restricted it to the Eriococcidae. Currently, it is accepted that this genus belongs to the Kermesidae (Morrison and Morrison 1966, Bullington and Kosztarab 1985, Baer and Kosztarab 1985, Ben-Dov et al. 2012).

Most species of the Kermesidae appear to be univoltine (Balachowsky 1950, 1953, McConnell and Davidson 1959, Hamon et al. 1976, Sternlicht 1969, Koteja 1980, Bullington and Kosztarab 1985, Hu 1986, Kosztarab and Kozár 1988, Viggiani 1991, Liu et al. 1997, Marotta et al. 1999). Females develop through three nymphal instars before reaching maturity (Bullington and Kosztarab 1985). Males are only known for a few species and the remaining species are thought to reproduce parthenogenetically (McConnell and Davidson 1959, Sternlicht 1969, Koteja and Zak-Ogaza 1972, Hamon et al. 1976, Baer and Kosztarab 1985, Hu 1986, Kosztarab and Kozár 1988, Miller and Miller 1993, Liu et al. 1997, Marotta et al. 1999, Turner 2004). Kermesidae are oviparous and, after the female’s last molt and before oviposition, the scale’s body increases its size and the dorsum becomes convex and sclerotized. The female lays eggs in the brood chamber which is located beneath the female’s venter (Bullington and Kosztarab 1985). The size, shape, and color pattern of post-reproductive females varies considerably within the same species (Bullington and Kosztarb 1985).

The body of post-reproducing females may remain on the host tree for a year or more after the emergence of the first-instar nymphs (Baer 1980, authors’ observations). Most species of Kermesidae are not known to cause any visible injury to their host trees although there are reports of branch dieback, flagging, reduced growth rates and occasional tree death (Kozár 1974, Hamon 1977, Solomon et al. 1980, Viggiani 1991, Pellizzari et al. 2012). On the other hand, some species of *Kermes* are known for their importance as a source of crimson dye (Amar 2005, Cardon 2007).

The main synapomorphic characters of kermesid adult females have been summarized by several authors including: Ferris (1955), Bullington and Kosztarab (1985), Hodgson (1997) and Miller et al. (2005). They are the presence of: (i) bilocular pores on venter; (ii) simple pores on dorsum; (iii) tubular ducts; (iv) a three-segmented labium with setae; (v) a group of multilocular disc-pores near the base of each antenna; (vi) an anal ring with or without cells and (vii) anal ring with or without setae and absence of: (viii) a spiracular band of quinquelocular disc-pores, (ix) stigmatic spines and (x) ventral microducts.

Species of *Nidularia* share morphological and biological characteristics with other species of the Kermesidae. Like *Kermes* spp., *Nidularia* spp. are monophagous and develop on oak trees. They are known as ‘gall-like insects’ due to the size and body shape of the convex and sclerotized post-reproductive adult female (Kosztarab and Kozár 1988). They mainly develop in bark crevices, forks between small twigs and buds, and on branches (Bullington and Kosztarab 1985).

Seven species of Kermesidae belonging to two genera, *Kermes* Boitard and *Nidularia* Targioni-Tozzetti, are recorded from Israel (Ben-Dov et al. 2012). Two species, namely *Kermes greeni* and *Kermes nahalali*, were originally described from post-reproductive adult females (Bodenheimer 1931), three species: *Kermes echinatus*, *Kermes palestiniensis* and *Kermes spatulatus* were described from the first-instar nymphs (Balachowsky 1953), and one species, *Kermes bytinskii*, was described from the adult female and all nymphal instars by Sternlicht (1969). *Nidularia balachowskii* was originally described from Turkey (Bodenheimer 1941), and then, in the same year, it was collected in northern Israel at Daphne Oaks, which is today known as Horshat Tal Nature Reserve. Until now, the material collected from Daphne Oaks was the only known record of *Nidularia balachowskii* in Israel.

Between 2010 and 2012, we surveyed the scale insect fauna of various species of oak trees in Israel. *Nidularia balachowskii* was found on branches of *Quercus ithaburensis* over a wide range of oak forests in northern Israel. Although Hoy (1963) and Koteja (1980) recorded *Nidularia pulvinata* from Israel, we did not encounter this species during our forest surveys. In addition, no material of *Nidularia pulvinata* from Israel was found among the Kermesidae records of dry material and microscope slides collections examined at the MNHN, BMNH, TAU and ICVI. To date it seems that *Nidularia balachowskii* is the sole *Nidularia* species present in Israel.

Bodenheimer’s original description of *Nidularia balachowskii* contains a description and some illustrations of major characters in the adult female (Bodenheimer 1941). His description is short and incomplete. The main objective of this paper is to redescribe *Nidularia balachowskii* and to establish its generic and family placement. Our descriptions and illustrations are based on the lectotype and paralectotype specimens (see Material examined) as well as on fresh material collected from Israel and Turkey. In addition, the first-instar nymph is described and illustrated for the first time. An identification key for the first-instar nymphs of *Nidularia* is provided. We also compare some characters of the adult female of *Nidularia balachowskii* with those of several other species of Kermesidae: *Nidularia pulvinata*, *Kermes roboris* (Fourcroy) (type species of *Kermes*) and *Kermes quercus* (Linnaeus). In addition, sequences of 28S and COI genes from *Nidularia balachowskii* and five or six other kermesids species plus some scale insect representatives of other Coccoidea families were compared in order to elucidate the family placement *of N. balachowskii*. Information on habitat and life history of *Nidularia balachowskii* in Israel is also provided.

## Material and methods

### Specimen collections

This redescription of *Nidularia balachowskii* is based on type material (see Material examined below), plus specimens from Israel collected by Bodenheimer and fresh material collected in Israel by us. Populations of *Nidularia balachowskii* from *Quercus ithaburensis* trees were studied and specimens were collected between 2010 and 2012 from the following nature reserves in northern Israel: Yehudiya Nature Reserve, Golan Heights (32°56'19"N, 35°39'56"E﻿); Horshat Tal Nature Reserve, Upper Galilee (33°13'13.74"N, 35°37'45.65"E); Alonei Abba Nature Reserve, Lower Galilee (32°43'46.2"N, 35°10'18.47"E). Trees at each reserve were surveyed at least once a month and 150–200 branches (20–25 cm in length) were removed at each visit. The branches were taken back to the laboratory in large plastic bags and examined individually under a stereomicroscope for scale insects. Relevant specimens were slide-mounted for microscope examination using the protocol in Ben-Dov, Hodgson (1997). Specimens of *Nidularia pulvinata* (adult females and first-instar nymphs) became available from MNHN. Material of *Nidularia japonica* was not available and comparisons with this species were based on the original description by Kuwana (1918) as well on the redescription by Liu et al. (1997). Dry and mounted material of *Nidularia balachowskii* from Israel, are deposited in the ICVI, BMNH and MNHN.

### Identification and morphological observations

Illustrations of the adult female and the first-instar nymph of *Nidularia balachowskii* are generalizations of several specimens, showing the dorsum on the left and the venter on the right, with enlargements of important structures arranged around the main drawing. The enlarged structures are not drawn to the same scale. Terms for morphological features follow chiefly those of Bullington and Kosztarab (1985), Baer and Kosztarab (1985) and Hodgson (1994). Measurements of specimens and of morphological structures were made using an ocular micrometer on an Olympus BX51 phase contrast microscope. Measurements of structures are given in microns (µm) and millimeters (mm). Body length was measured from the farthest points of the head to the posterior end of the body, and body width was the greatest width. Setae lengths were measured from the tip of its base (excluding the setal socket) to the apical tip of the setae. The frequency of each structure is given for the entire body. The range is taken from twenty specimens.

Abbreviations of specimen depositories are as follows: **BMNH** -The Natural History Museum, London, U.K.; **ICVI** - Coccoidea Collection, Department of Entomology, Agricultural Research Organization, Bet Dagan, Israel; **MNHN** - Museum National d’ Histoire Naturelle, Paris, France; and **TAU** - Tel Aviv University Insect Collection, Israel.

## Material examined

### *Nidularia balachowskii*.

**Turkey**: **Lectotype** female (ICVI), here designated, and **paralectotype** female (MNHN), 21 km at road from Mardin to Diyarbakir, on branches and twigs of *Quercus* sp. (Fagaceae), 13.ii.1939, F.S. Bodenheimer. Bodenheimer (1941) did not select a holotype, and we regard the above-mentioned specimens as the original material studied by him as indicated on the slide labels.

**Additional non-type material** from **Turkey** as follows: Van-Koçet Road (alt.1625 m) on *Quercus* sp., 19.vii.2005, B. Kaydan (Yuzuncu Yil University, Turkey 2056); Hakkari -Üzümcü Road (alt. 956 m) on *Quercus* sp., 15.ix.2005, B. Kaydan (Yuzuncu Yil Universty, Turkey 2343); Van-Hakkari Road (alt. 1266 m) on *Quercus* sp., 16.ix.2005, B. Kaydan (Yuzuncu Yil Universty, Turkey 2370); Hakkari-Doğan (alt. 1032 m) on *Quercus* sp., 22.v.2005, B. Kaydan (Yuzuncu Yil Universty, Turkey 2688); Bitlis River (alt. 797 m) on *Quercus sp*., 23.vi.2006, B. Kaydan (Yuzuncu Yil Universty, Turkey 3036); Bitlis-Kavakbaşı (alt. 1365) on *Quercus* sp. 30.v.2007, B. Kaydan (Yuzuncu Yil Universty, Turkey 3419).

**Israel:** adult female, Daphne Oaks (= current name Horshat Tal Nature Reserve), on *Quercus* sp., 1.v.1939, F.S. Bodenheimer, (ICVI C:4805). This was the first record of this species from Israel. Additional females and first-instar nymphs, all collected off *Quercus ithaburensis* by M. Spodek: Yehudiya Nature Reserve, 10.x.2010, 7.xi.2010, 11.i.2011, 6.ii.2011, 16.x.2011, 6.xi.2011, 3.vi.2012, (ICVI C:4891, C:4912, C:4945, C:4970, MC:587, C:4970, MC:690); Horshat Tal Nature Reserve, 30.v.2010, 14.ii.2012, 27.ii.2011, 13.iii. 2012, (ICVI MC:228, MC:614, MC:430, C:5131); Alonei Abba Nature Reserve, 11.1.2011, (ICVI MC:385). First-instar nymphs; Yehudiya Nature Reserve, 6.iii.2010, 20.iii.2011, 24.iii.2012, (ICVI MC:140, MC:460, MC:637); Horshat Tal Nature Reserve, 13.iii.2012, 15.iii.2012, (ICVI MC:635, MC:636).

**Comparative material examined**

***Nidularia pulvinata***: **France**: adult female and first-instar nymphs, Serignan, Vauctuse, on *Quercus ilex*, 18.v.1978, I. Foldi (ICVI C:4946); first-instar nymphs, Caumont (Avignon), on *Quercus ilex*, 11.iv.1978, D. Matile, J.P. Fabre, (MNHN 7337-4); **Italy**: adult femalePisa, on *Quercus ilex*, 5.iv.1988, D. Matile-Ferrero (MNHN10959, ICVI 5060)

***Kermes quercus***: **Sweden**: adult female, Skan, Near Lund on *Quercus robur*, 10.vi.2010, C.A Gertsson (ICVI C:4806); **England**: adult female , Wytham Wood, Berkshire on *Quercus robur*, 10.v.1965, S.C. Varley (BMNH 81-539); **Poland**; first-instar nymph, Warsaw on *Quercus robur*, 25.viii.1994, E. Podsiadlo (ICVI C:4798)

***Kermes roboris***: **Hungary**: adult female, Budapest, Plant Protection Institute-adjacent track on *Quercus* sp., 8.vi.1989, C.P. Malumphy (BMNH); **England**: adult female, Kent: Herne Bay on *Quercus* spp., 00.viii.1899, C.D Waterhouse (ICIV C:5071).

## Molecular methods

### Samples

Specimens of the following adult female kermesidspecies, identified by YBD, were used in the molecular part of this study: *Kermes nahalali* Bodenheimer, *Kermes echinatus* Balachowsky, *Kermes greeni* Bodenheimer, *Kermes quercus* (Linnaeus), *Kermes spatulatus* Balachowskyand *Nidularia balachowskii*. Three specimens of each species were used as replicates except for *Nidularia balachowskii*, where six were used. Adult females, preserved in 96% ethanol, were examined under the stereomicroscope for the presence of hymenopteran parasitoid wasps prior to DNA extraction. Voucher specimens were slide-mounted using the cuticle of the actual specimens from which DNA was extracted. Slide mounting followed the protocol outlined in Ben-Dov, Hodgson (1997), and the voucher slides are deposited in the ICVI (Table 1). To provide some taxonomic context to our study, we included also DNA sequences of species belonging to other families within the Coccoidea: Asterolecaniidae, Coccidae, Diaspididae, Eriococcidae, Monophlebidae and Pseudococcidae. A species of aphid, *Acyrthosiphon pisum* (Hemiptera, Aphidoidea), was used as the outgroup species. These sequences were made available from GenBank (Table 1). The sequences of Kermesidae species obtained in this study are deposited in the GenBank under the accession numbers JX436113 - JX436154.

**Table 1.** Collection information and GenBank accession numbers for insect samples used in this study.

**Species name**	**Family**	**Voucher code**	**Host tree**	**Location collected**	**Date collected**	**Collector**	**GenBank Accession No. 28S**	**GenBank Accession No. COI**
*Kermes nahalali* Bodenheimer	Kermesidae	C-5111	*Quercus ithaburensis*	ISRAEL: Alonei Abba Reserve	27.ii.2011	M. Spodek	JX436134	JX436113
*Kermes nahalali* Bodenheimer	Kermesidae	C-5112	*Quercus ithaburensis*	ISRAEL: Alonei Abba Reserve	27.ii.2011	M. Spodek	JX436135	JX436114
*Kermes nahalali* Bodenheimer	Kermesidae	C-5113	*Quercus ithaburensis*	ISRAEL: Alonei Abba Reserve	27.ii.2011	M. Spodek	JX436136	JX436115
*Kermes echinatus* Balachowsky	Kermesidae	C-5114	*Quercus calliprinos*	ISRAEL: Alonei Abba Reserve	19.vi.2011	M. Spodek	JX436137	JX436116
*Kermes echinatus* Balachowsky	Kermesidae	C-5115	*Quercus calliprinos*	ISRAEL: Alonei Abba Reserve	19.vi.2011	M. Spodek	JX436138	JX436117
*Kermes echinatus* Balachowsky	Kermesidae	C-5116	*Quercus calliprinos*	ISRAEL: Alonei Abba Reserve	19.vi.2011	M. Spodek	JX436139	JX436118
*Kermes greeni* Bodenheimer	Kermesidae	C-5117	*Quercus calliprinos*	ISRAEL: Hanita	8.vi.2011	M. Spodek	JX436140	JX436119
*Kermes greeni* Bodenheimer	Kermesidae	C-5118	*Quercus calliprinos*	ISRAEL: Hanita	8.vi.2011	M. Spodek	JX436141	JX436120
*Kermes greeni* Bodenheimer	Kermesidae	C-5119	*Quercus calliprinos*	ISRAEL: Hanita	8.vi.2011	M. Spodek	JX436142	JX436121
*Kermes quercus* (Linnaeus)	Kermesidae	C-5120	*Quercus robur*	SWEDEN: Skan, near Lund	10.vi.2010	C. A. Gertsson	JX436143	JX436122
*Kermes quercus*(Linnaeus)	Kermesidae	C-5121	*Quercus robur*	SWEDEN: Skan, near Lund	10.vi.2010	C. A. Gertsson	JX436144	JX436123
*Kermes quercus* (Linnaeus)	Kermesidae	C-5122	*Quercus robur*	SWEDEN: Skan, near Lund	10.vi.2010	C. A. Gertsson	JX436145	JX436124
*Kermes spatulatus* Balachowsky	Kermesidae	C-5123	*Quercus ithaburensis*	ISRAEL: Horshat Tal Reserve	3.iv.2011	M. Spodek	JX436146	JX436125
*Kermes spatulatus* Balachowsky	Kermesidae	C-5124	*Quercus ithaburensis*	ISRAEL: Horshat Tal Reserve	3.iv.2011	M. Spodek	JX436147	JX436126
*Kermes spatulatus* Balachowsky	Kermesidae	C-5125	*Quercus ithaburensis*	ISRAEL: Horshat Tal Reserve	3.iv.2011	M. Spodek	JX436148	JX436127
*Nidularia balachowskii* Bodenheimer	Kermesidae	C-5126	*Quercusithaburensis*	ISRAEL: Yehudiya Reserve	11.i.2011	M. Spodek	JX436149	JX436128
*Nidularia balachowskii* Bodenheimer	Kermesidae	C-5127	*Quercus ithaburensis*	ISRAEL: Yehudiya Reserve	11.i.2011	M. Spodek	JX436150	JX436129
*Nidularia balachowskii* Bodenheimer	Kermesidae	C-5128	*Quercus ithaburensis*	ISRAEL: Alonei Abba Reserve	27.ii.2011	M. Spodek	JX436151	JX436130
*Nidularia balachowskii* Bodenheimer	Kermesidae	C-5129	*Quercus ithaburensis*	ISRAEL: Alonei Abba Reserve	27.ii.2011	M. Spodek	JX436152	JX436131
*Nidularia balachowskii* Bodenheimer	Kermesidae	C-5130	*Quercus ithaburensis*	ISRAEL: Horshat Tal Reserve	14.ii.2012	M. Spodek	JX436153	JX436132
*Nidularia balachowskii* Bodenheimer	Kermesidae	C-5131	*Quercus ithaburensis*	ISRAEL: Horshat Tal Reserve	14.ii.2012	M. Spodek	JX436154	JX436133
*Kermes nakagawae* Kuwana	Kermesidae						n/a	AB439525.1
*Bambusaspis miliaris* (Boisduval)	Asterolecaniidae						GU998966.1	n/a
*Ceroplastes rubens* Maskell	Coccidae						n/a	JQ795720.1
*Paralecanium* sp.	Coccidae						GU998968.1	n/a
*Pelliculaspis celtis* McDaniel	Diaspididae						GQ325525.1	n/a
*Apiomorpha nookara* Mills	Eriococcidae						n/a	JN863289.1
*Eriococcus spurius* (Modeer)	Eriococcidae						GU998969.1	n/a
*Drosicha mangiferae* (Green)	Monophlebidae						n/a	JF792882.1
*Phenacoccus parvus* Morrison	Pseudococcidae						n/a	JQ863289.1
**Outgroup**								
*Acyrthosiphonpisum* Harris	Aphididae						S50426.1	EU701281.1

### DNA extraction, amplification and sequencing

DNA was extracted from parasitoid-free adult females using the Cetyl trimethylammonium bromide (CTAB) method (Murray and Thompson 1980). Polymerase chain reaction (PCR) products were generated from the mitochondrial Cytochrome Oxidase I (COI) gene, and a fragment of the D2 and D3 regions of the 28S ribosomal DNA gene. PCR reaction was performed in a total volume of 25 µL containing 1 unit of dream Taq polymerase (Fermentas, USA), 2.5 µL of enzyme buffer supplemented with MgCl2, 0.2 µL of 25 mM dNTPs, 0.3 µL of 20 pmole for each primer, and 2 µL of DNA template. A 900 bp fragment of the 28S ribosomal RNA gene and a 400 bp fragment of the COI gene were amplified and sequenced. Primers for both genes were 28S forward 5’-GAC CCG TCT TGA AAC ACG GA-3’ and 28S reverse 5’-TCG GAA GGA ACC AGC TAC TA-3’ (Gullan et al. 2010). COI forward 5’-CAA CAT TTA TTT TGA TTT TTT GG-3’ (C1-J-2183 aka Jerry) and COI reverse 5’-GCW ACW ACR TAT AKG TAT CAT G-3’ (C1-N-2568 aka Ben) (Gullan et al. 2010). The COI barcode region (Herbert et al. 2003) was not used because it has failed to amplify in most scale insects tried to date (Schroer et al. 2008).

The PCR cycling conditions for 28S were 94°C for 4 min, followed by 35 cycles of 94°C for 1 min, 50°C for 1 min, and 72°C for 1.5 min, with a final extension at 72°C for 4 min. The PCR cycling protocol for COI was 95°C for 7 min, followed by 40 cycles of 95°C for 1 min, 45°C for 1 min, and 72°C for 1.5 min, with a final extension at 72°C for 5 min. Each reaction was examined by electrophoresis and bands were visualized with UV light. PCR products were excised from the gel and purified using the Zymoclean Gel Extraction Kit (Zymo Research, Irvine, CA). Purified PCR products were sequenced in both the forward and reverse directions at Hy-Labs (Rehovot, Israel).

### Sequence alignment and phylogenetic analysis

Sequence alignments for both 28S and COI gene sequences were performed with MUSCLE 3.7 (Edgar 2004) and the results were adjusted manually where necessary to maximize alignment. The alignment data for each gene were used in maximum likelihood tree construction, using Kimura-2 parameter model (K2P) genetic distances (Kimura 1980). Both trees were generated using MEGA v.5 (Tamura et al. 2011) and branch support was estimated with 1000 bootstrap replicates.

## Results

### Morphological characteristics

#### Nidularia balachowskii

Bodenheimer

http://species-id.net/wiki/Nidularia_balachowskii

Nidularia balachowskii Bodenheimer, 1941: 78–80.

##### Adult female.

**Figure 1.**
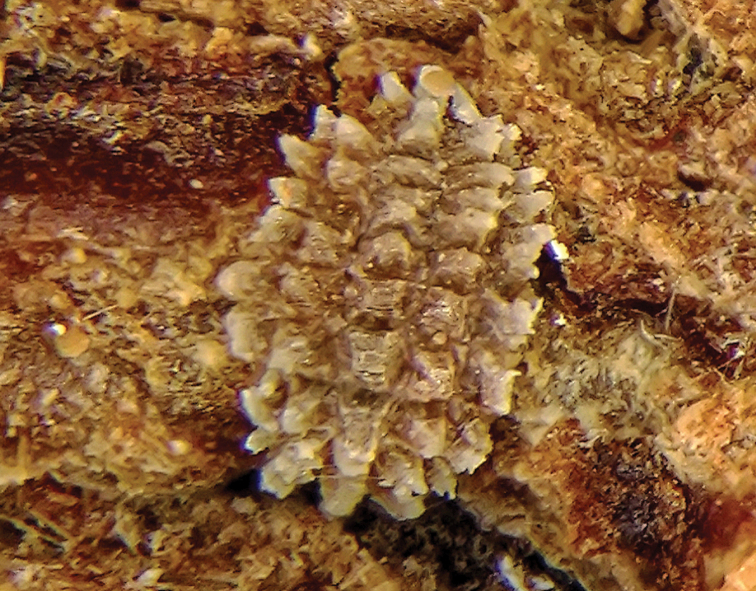
*Nidularia balachowskii* Bodenheimer young adult female, general appearance.

**Figure 2.**
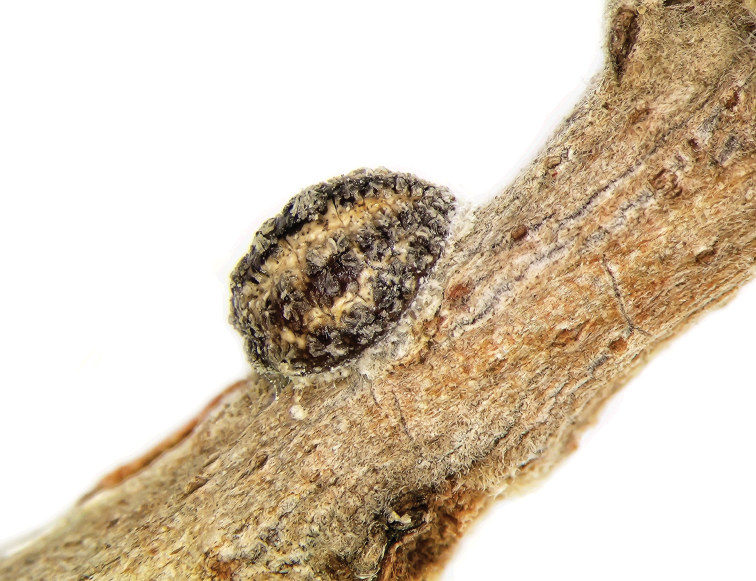
*Nidularia balachowskii* Bodenheimer post-reproductive female, general appearance.

**General appearance.** **Young, pre-reproductive adult** dorsum brownish and venter yellowish white; oval, soft and flat; 1.2–1.9 mm long and 0.6–0.9 mm wide. Dorsal surface covered with 5 longitudinal rows of rectangular wax plates, each plate about 0.25 mm long and 0.3 mm wide; median row with 11 plates, lateral row on each side of median row with 9–11 plates and marginal rows with 7–9 plates. The wax plates become gradually smaller in size towards anterior and posterior apices and lateral margin (Fig. 1). **Post-reproductive female** oval, moderately convex and sclerotized; 2.75–3.75 mm long, 2–3 mm wide and 0.8–1.8 mm high; 5 longitudinal rows of dark brown wax plates almost fused; with lighter brown wax in between rows of plates (Fig. 2).

**Figure 3.**
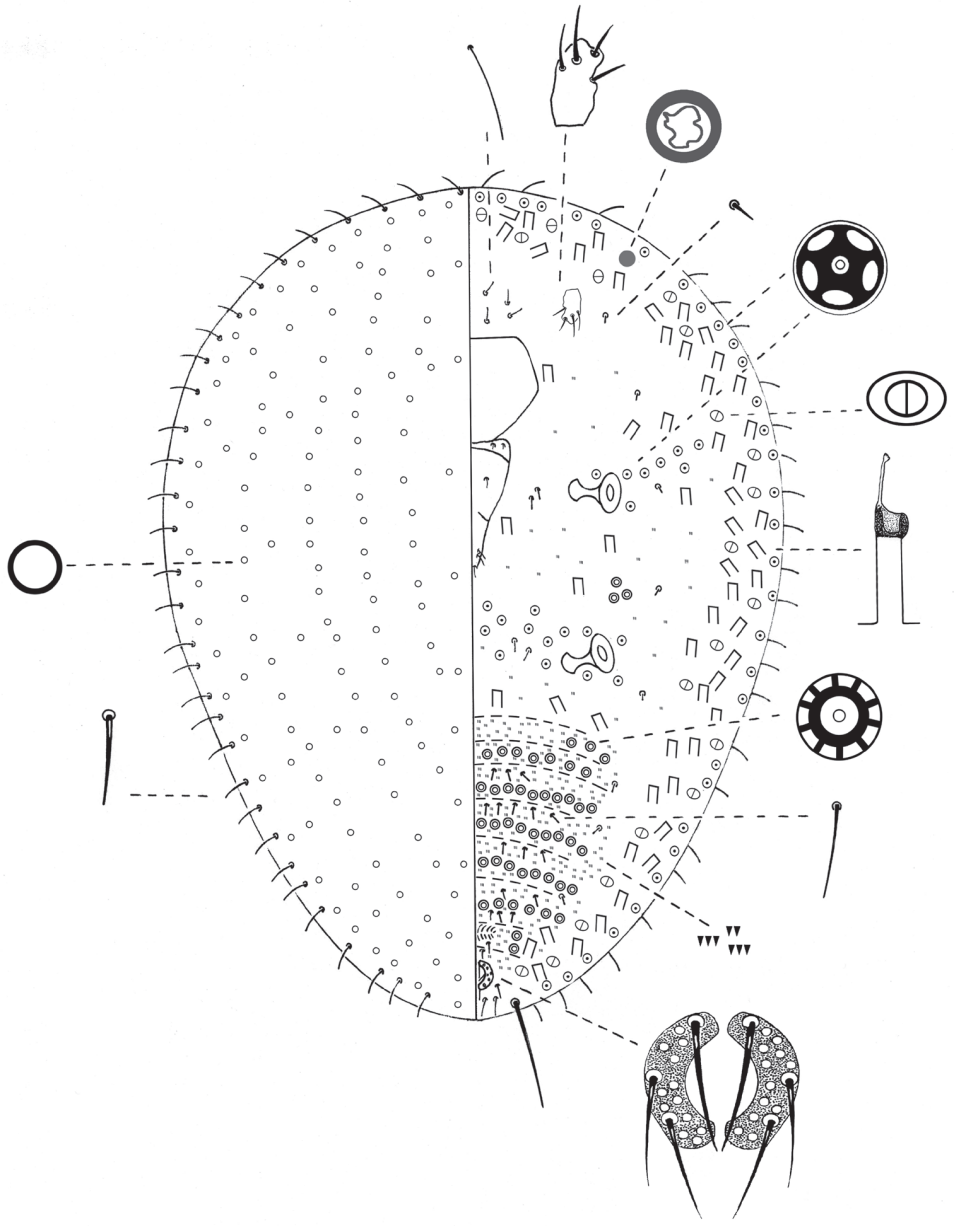
*Nidularia balachowskii* Bodenheimer adult female.

**Slide-mounted** **young** **adult female** 0.8– 3 mm long, 0.5–2.5 mm wide (Fig. 3).

**Margin. Marginal setae,** pointed, 12–13 µm long; placed in a row of 30–36 setae. Stigmatic spines absent. Anal cleft absent.

**Dorsum. Simple pores**, circular, each with a sclerotized rim and 1 µm diameter; covering entire dorsum. Other pore types absent. Dorsal setae absent.

**Venter. Eyes** circular, 5–7 µm diameter, each placed anterolaterally to each antenna. **Antennae** each1-segmented, 15–25 µm long, 12–18 µm wide**;** eachbearing 2–6 fleshy setae. **Legs** absent. **Clypeolabral shield** 113–155 µm long, 113–125 µm wide. **Labium** 3-segmented, triangular, 100–125 µm long, 50–63 µm wide; labial setae as follows; basal segment with 2 pairs of hair-like setae, 9–20 µm long, median segment with 1 pair of hair-like setae, 11–13 µm long, apical segment with 6 setae; 2 apical setae, 9–12 µm long plus 4 hair-like; subapical setae, each 12–20 µm long. **Spiracles** subequal in size; each 42–60 µm long, 31–50 µm diameter of peritreme. **Quinquelocular pores** each 5 µm diameter; with 8–11 between mesothoracic spiracles and submarginal band of tubular ducts; 10–13 between the metathoracic spiracles; 2–4 laterad to each metathoracic spiracle; also in a single, complete submarginal band, 1 pore wide from head apex to anal ring; total number of pores per side about 50–71.**Bilocular pores** each about 3 µm wide, totaling 103–135 per side, dispersed within a submarginal tubular duct band. **Multilocular pores** each 7–8 µm in diameter with 9–10 loculi; in groups of 3 or 4 between each metathoracic and mesothoracic spiracle;in transverse bands across abdominal segments arranged as follows; segment III with 3–7, IV with 12–24, V with 14–28, VI with 15–35, VII with 18–25, VIII with 12–19, IX with 2–3 on each side of vulva. **Tubular ducts** dispersed in a complete submarginal band, 2–3 ducts wide, each duct with outer ductule 12–18 µm long and 5 µm wide, a sclerotized cup about 5 µm in diameter and inner ductule about 22–30 µm long; also scattered over thorax. **Other ventral setae:** with a group of 7 or 8 setae, each 7–13 µm long, anterior to clypeus; 1 pair, 7–8 µm long, posterior to each antenna; 8–12 setae, each 5–8 µm long, placed medially to each spiracle; 8 setae, 5–13 µm long, distributed in 1 longitudinal row placed medially to each marginal band of bilocular pores and tubular ducts; each abdominal segment with transverse rows of 4–10 setae, each 7–8 µm long, placed anterior to bands of multilocular pores. **Microspines** present on median and submedian areas of each abdominal segment, in 3–5 transverse rows; each spine about 1 µm long, also scattered on thorax. **Anal ring** located on venter,composed of 2semi-circles; diameter27–35 µm; each half circle bearing 3 pointed setae and 10–12 cells; anterior setae each 25–38 µm long, median and posterior setae each 15–25 µm long; 2 pairs of thin setae just anterior to anal ring, each 7–10 µm long, plus a pair of pointed setae postero-laterally to anal ring, separated by a space about double diameter of anal ring, each 10–13 µm long. Also1 pair of apical setae, each 65–68 µm long, and 4 setae, each 15–22 µm long, between apical setae, similar in structure to, but longer than, marginal setae.

##### First-instar nymph.

**General appearance.** Yellow-greyish, oval and tapering posteriorly 0.38–0.43 mm long and 0.2–0.3 mm wide.

**Figure 4.**
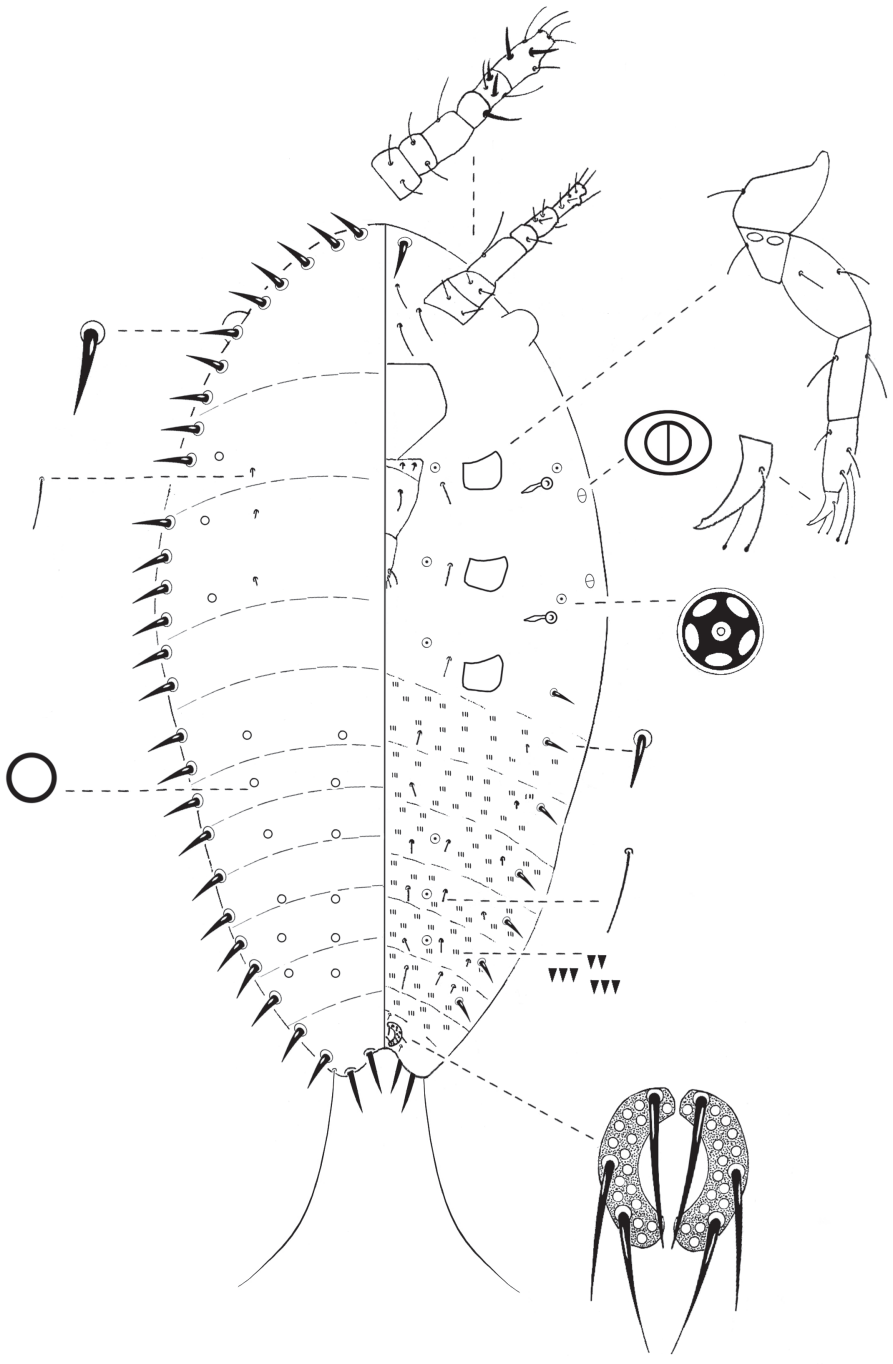
*Nidularia balachowskii* Bodenheimer first-instar nymph.

**Slide-mounted specimen**. Oval, 0.42–0.53 mm long and 0.19–0.34 mm wide (Fig. 4)**.**

**Margin. Marginal setae** sharply spinose, pointed apically and slightly curved; each 9–15 µm long; in a distinct row of 26–33 setae on each margin.

**Dorsum.** Derm membranous; intersegmental lines observable. **Simple pores**, circular with a sclerotized rim, each 1 µm diameter; totaling about 30, scattered over entire dorsum in 4 longitudinal rows; 2 submarginal lines on thorax and abdomen and 2 medial lines on abdomen; with a single pair of submedial setae on each thoracic segment, each 5 µm long.

**Venter. Eyes** present as semi-circles near margin, diameter 12–15 µm. **Antennae** each 6-segmented, length 88–125 µm; segments III and VI longer than other segments; scape with 2 hair-like setae; pedicel with 2 hair-like setae; segment III with 1 long hair-like seta; IV with 1 fleshy seta; V with 1 fleshy seta, 2 hair-like setae and, 1 thick hair-like seta; apical segment with 2 fleshy setae and 5 hair-like setae. **Legs** well-developed; measurements of hind legs; (in µm): coxae 25–30, trochanter + femur 63–80, tibia 25–38, tarsus 25–60, claw 13–23; total leg length 158–213; trochanter with 2 oval, sensory pores on each side, each about 2–3 µm wide; setae present on each leg segment; tarsal digitules each 25–30 µm long, knobbed apically, extending beyond apex of claw; claw digitules knobbed apically, each 14–20 µm long, shorter than tarsal digitules; each claw with a single denticle near the tip. **Clypeolabral shield** well-developed; 75–90 µm long and 63–75 µm wide. **Labium** 3-segmented, triangular, 82–100 µm long and 35–38 µm wide; labial setae as follows; basal segment with 2 pairs of setae, each 10–15 µm long, median segment with 1 pair of hair-like setae on dorsal surface, each 10–20 µm long, apical segment with 3 pairs of hair-like setae, 12–17 µm long. **Spiracles** subequal in size; peritreme about 3–5 µm diameter; crescent shaped sclerosis 15–25 µm long. **Bilocular pores**, oval, each 2 µm long and 1 µm wide, with 1 present submarginally about level of each spiracle. **Quinquelocular pores** each 3 µm diameter, as follows; 1 just anterior to each spiracle; 1 medially to each coxa; 2 on each of abdominal segments IV, V, VI. **Microspines** present on median and submedian areas of each abdominal segment, in 2–4 transverse rows; each spine about 1 µm long. **Setae** 1 pair, similar in size and shape to marginal setae, between anterior apex of body and basal segments of antennae; 6 interantennal setae, each 20–30 µm long between basal segments of antennae and anterior apex of clypeus; 1 seta 8–15 µm long medially to each coxa; 2 longitudinal rows, each with 7–8 setae, similar in shape to marginal setae but shorter, each about 8 µm long, extending submarginally from laterad to metathoracic coxae to anterior margin of anal ring, no setae present on most posterior abdominal segment; 2 longitudinal rows of 6 setae, each about 5 µm long, located submarginally on abdomen; 2 longitudinal rows of 4 setae, each about 10 µm long, located submedially on abdomen; plus 2 longitudinal rows of 6 setae, about 17–18 µm long, located medially. **Anal ring** located ventrally; composed of 2 semi-circles; diameter 15–25 µm; each semi-circle with about 17 cells and 3 pointed setae, subequal in size, each 20–30 µm long. Also with a pair of setae, each 12–15 µm long, anterior to anal ring and 1 pair, each 15–25 µm long**,** postero-laterally to anal ring. **Anal lobes** well-developed; each lobe bearing 2 pointed setae, 12–15 µm long, distinctly thicker than marginal setae, plus 1 pair of long, apical setae 77–125 µm long.

### Results

Molecular identification and relationships

Molecular results

**Figure 5.**
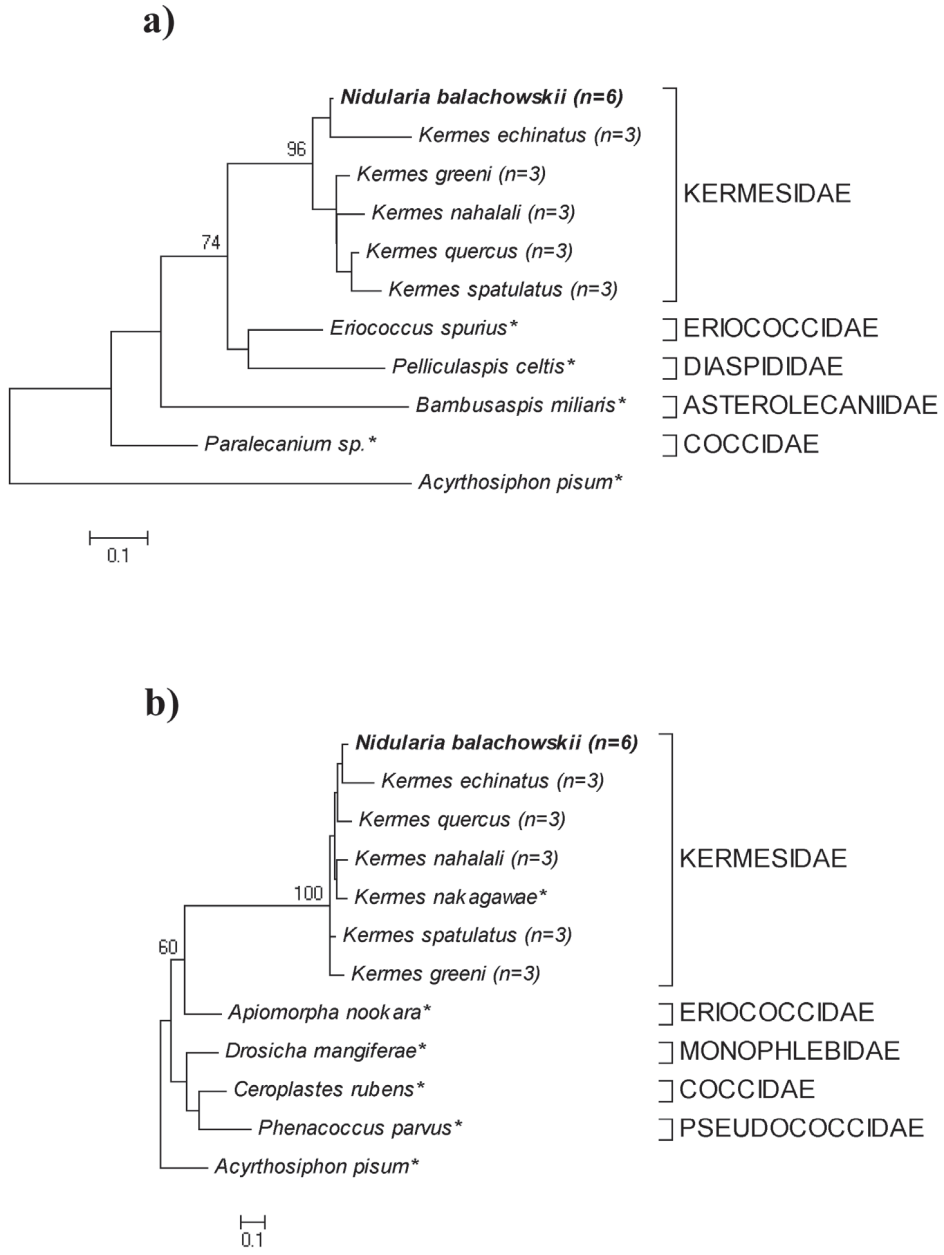
Maximum likelihood trees of 28S (**a**) and COI (**b**) nucleotide sequences of *Nidularia balachowskii* and other Coccoidea species. *Acyrthosiphon pisum* (Aphididae) sequences are used as outgroup species for both trees. Trees were constructed using K2P distance model and numerical values are bootstrap support, based on 1000 replicates (n= number of replicates, * = sequences derived from GenBank).

We obtained a total of forty-two nucleotide sequences from the 28S and COI genes from *Nidularia balachowskii* (six individuals for each gene) and from five adult female Palaearctic Kermesidae species (three individuals for each species for each gene). 28S gene sequences (~700 bp) and COI sequences (~400 bp) from all species were recovered and aligned with sequences of Coccoidea species representing different families (obtained from GenBank). All species for which multiple specimens were sampled showed no interspecies variation. The maximum likelihood analysis of both genes resulted in tree typologies that show that *Nidularia balachowskii* is a distinct species within the monophyletic Kermesidae. *Nidularia balachowskii* is grouped together with other kermesid species and not with the other Coccoidea (Figures 5a+b). The bootstrap value that represents the separation between species of Kermesidae and species from other Coccoidea families is higher in the 28S tree typology, 74, compared to 60 obtained from the COI sequences.

Sequence divergence based on Kimura 2-parameter pairwise distance, between *Nidularia balachowskii* and the other five Kermesidae species ranged from 0.16–0.19 in the 28S gene region. This range is compared to the 0.2–0.3 sequence divergence range between *Nidularia balachowskii* and species from the four other Coccoidea families. In the COI gene region, the sequence divergence between *Nidularia balachowskii* and the six other Kermesidae species ranged from 0.06–0.13 and between *Nidularia balachowskii* and the four species from other Coccoidea families had a sequence divergence range of 0.8–1. Both trees show a strong relationship between *Nidularia balachowskii* and *Kermes echinatus*, indicating that they are closer to each other than to the other Kermesidae species examined.

### Life history

Observations about the life history of *Nidularia balachowskii* were made in three nature reserves in northern Israel: Alonei Abba Nature Reserve, Horshat Tal Nature Reserve and Yehudiya Nature Reserve. The predominant oak species growing in these reserves is *Quercus ithaburensis*. In Alonei Abba Reserve, *Quercus calliprinos* trees are also present but they are less common. In Israel, *Nidularia balachowskii* has only been found on the trunks and branches of *Quercus ithaburensis*, where *Nidularia balachowskii* is an oviparous and univoltine species.

Gravid females were observed on branches and trunks of trees throughout March, during which time they oviposited 200 to 250 (range from 10 specimens) whitish eggs. Each egg was about 0.4 mm long and 0.2 mm wide. Once all of the eggs have been laid and the brood chamber full of eggs, the female dies and the dorsum becomes sclerotized. The sclerotized, convex body of the dead, post-reproductive female may remain on the host tree for a year or more after first-instar emergence.

Eclosion of first-instar nymphs occurs inside the brood chamber and nymphs emerge from the cavity under the dead female body. This takes place from end of March and throughout April. Crawlers settle in bark crevices on branches and on the trunks of the trees. Young teneral females are found on the branches from June to February. The females continue feeding and increase in body size throughout this period. Feeding was confirmed by observations of honeydew elimination. By late February, the dorsum of the female begins to expand greatly, increasing in convexity and sclerotization. The ventral surface of the abdomen becomes concave, forming the brood chamber into which the eggs are deposited. The ovipositing female secretes a woolly, white wax that surrounds its body margin. No injury has been observed to the oak hosts by *Nidularia balachowskii* in Israel.

## Discussion

### Life history

We compared our observations of the host plant and development of *Nidularia balachowskii* in Israel to Bodenheimer’s 1941 records of this species. In Israel, this scale insect has only been found on the trunks and branches of *Quercus ithaburensis* trees, whereas Bodenheimer gives *Quercus* sp. as the host tree in Turkey (Bodenheimer 1941) and Iran (Bodenheimer 1944). The geographical distribution of *Quercus ithaburensis* is wide, extending also to Turkey and Iran, and so we may speculate that Bodenheimer’s *Quercus* sp. is probably *Quercus ithaburensis* ssp. *macrolepis* (Dufour-Dror and Ertas 2004).

We observed that *Nidularia balachowskii* is an oviparous, univoltine species in Israel, similar to Bodenheimer’s (1941) observations in Turkey. Earlier observation on the other two species of *Nidularia* indicatedthat both *Nidularia pulvinata* in Italy (Viggiani 1991) and *Nidularia japonica* in China (Liu et al. 1997) are univoltine. All three species of *Nidularia* have only been recorded so far on oak trees (Ben-Dov et al. 2012). Koteja (1980) redescribed *Nidularia pulvinata* and noted that young specimens were covered with a fragile layer of wax and that, during expansion of the dorsum, this layer breaks into pieces and the females then secrete a nest-like ovisac ventrally and laterally. Kuwana (1918) and Liu et al. (1997) both describe a nest-like ovisac for female *Nidularia japonica*. In Israel, the teneral adult female of *Nidularia balachowskii* also produces a thin layer of dorsal wax but gravid females do not produce a nest-like ovisac. The eggs of *Nidularia balachowskii* are deposited into the egg cavity beneath the venter of the female, as described by Bullington and Kosztarab (1985) and Podsiadlo (2005a) for other kermesid species.

### Morphological characters of adult female

Some morphological characters of adult female *Nidularia balachowskii* are compared with those of *Nidularia pulvinata*, *Kermes quercus* and *Kermes roboris* (type species of *Kermes*) in Table 2, in order to evaluate the generic and family placement of the former species. All four species possess the following synapomorphic traits: three-segmented labium, bilocular pores on the venter, simple pores on the dorsum, quinquelocular pores surrounding the spiracles, and tubular ducts on the venter.These characters are some of the synapomorphic characters of kermesid adult females that have been described by Ferris (1955), Koteja (1980), Bullington and Kosztarab (1985) and Hodgson (1997).

Within the genus *Nidularia*, adult female *Nidularia balachowskii* share with *Nidularia pulvinata* the following characters: (i) one-segmented antennae; (ii) absence of legs; (iii) absence of setae-pore clusters on venter; (iv) ventral position of anal ring, and (v) an anal ring with setae and cells, whereas the two *Kermes* species have: four, five or six-segmented antennae, and possess legs, setae-pore clusters on venter, anal ring placed on dorsum, and an anal ring without setae and cells. Comparing *Nidularia balachowskii* and *Nidularia pulvinata*, the most obvious distinguishing feature is the presence of quinquelocular pores on the spiracle peritreme of *Nidularia pulvinata*.

**Table 2.** Morphological characters of adult females of *Nidularia balachowskii*, *Nidularia pulvinata*, *Kermes quercus* and *Kermes roboris*.

Character	Nidularia balachowskii	Nidularia pulvinata	Kermes quercus	Kermes roboris
antennal segments	1	1	4	5–6
labium segments	3	3	3	3
locular pores on spiracle peritreme	absent	present	absent	absent
locular pores surrounding spiracles	present	present	present	present
legs	absent	absent	present	present
bilocular pores on venter	present	present	present	present
simple pores on dorsum	present	present	present	present
setae -pore clusters on venter	absent	absent	present	present
tubular ducts on venter	1 type	1 type	2 types	2 types
anal ring location	ventral	ventral	dorsal	dorsal
anal ring shape	2 semi- circles	2 semi-circles	circular, complete	2 semi-circles
anal ring cells	present	present	absent	absent
anal ring setae	3 pairs setae	3 pairs setae	setae absent, rare with 2 setae	setae absent, rare with 2 setae

### Morphological characters of first-instar nymphs

The morphological characters of first-instar nymphs of Nearctic kermesids were summarized by Baer and Kosztarab (1985). Kuwana (1931) and Hu (1986) outlined some distinctive characters for separating first-instar nymphs of Oriental species of kermesids, and Balachowsky (1950, 1953) reviewed the characters of first-instar nymphs of some Palaearctic kermesids.

The first-instar nymphs of *Nidularia balachowskii* share the following characters with other Kermesidae species: (i) six-segmented antennae; (ii) three-segmented labium; (iii) simple pores forming longitudinal lines on the dorsum (iv) dorsal setae; (v) anal ring with cells and setae; (vi) microspines in rows on abdominal segments, and (vii) bilocular pores on venter. This last character is sometimes overlooked.

*Nidularia balachowskii* can be distinguished from other Kermesidae species by the form of its marginal setae. *Nidularia balachowskii* has sharply spinose, apically pointed and slightly curved setae, each 9–15 µm long. This differs from *Nidularia pulvinata* which has hair-like setae (Koteja 1980) and *Nidularia japonica* which has setose setae that are somewhat conical at the base (Kuwana 1918, Liu et al. 1997).The first-instars of Palaearctic *Kermes* species that have been described possess conical, hair-like or spatulate marginal setae (Kuwana 1931, Balachowsky 1950, 1953, Sternlicht 1969, Hu 1986, Liu et al. 1997, Podsiadlo 2005b, Pellizzari et al. 2012). There is no unique set of characters that distinguishes the first-instar nymphs of the genus *Nidularia* from the Palaearctic *Kermes* examined.

### Key to first-instar nymphs of *Nidularia* species*

**Table:** 

1	Quinquelocular pores absent on venter of abdomen	*Nidularia pulvinata* (Planchon)
–	Quinquelocular pores present on venter of abdomen	2
2	Marginal setae sharply spinose, apically pointed and slightly curved; quinquelocular pores, six, on venter of thorax only	*Nidularia balachowskii* Bodenheimer
–	Marginal setae setose and somewhat conical at base; quinquelocular pores, eight, on venter of head and thorax	*Nidularia japonica* Kuwana
* Characters of *Nidularia japonica* based on illustrations and descriptions by Kuwana (1918) and Liu et al. (1997).

### Molecular analysis

The DNA-sequence data for *Nidularia balachowskii*, six other species of Palaearctic Kermesidae, and species representing six other Coccoidea families showed that gene fragments of both COI and 28S separated *Nidularia balachowskii* from other Coccoidea species, and clearly placed *Nidularia balachowskii* in the Kermesidae. This study confirms that *Nidularia balachowskii* is a distinct species, clearly distinguishable from other closely-related kermesid species.

## Conclusion

Based on both the morphological and molecular studies of *Nidularia balachowskii*, the identity of *Nidularia balachowskii* and its placement in the Kermesidae has been substantiated, and we have shown that *Nidularia balachowskii* is congeneric with *Nidularia pulvinata*.

## Supplementary Material

XML Treatment for Nidularia balachowskii
